# Development and Validation of the User Version of the Mobile Application Rating Scale (uMARS)

**DOI:** 10.2196/mhealth.5849

**Published:** 2016-06-10

**Authors:** Stoyan R Stoyanov, Leanne Hides, David J Kavanagh, Hollie Wilson

**Affiliations:** ^1^ Centre for Children’s Health Research, Institute of Health & Biomedical Innovation School of Psychology and Counselling Queensland University of Technology (QUT) Brisbane, QLD Australia; ^2^ The Young and Well Cooperative Research Centre (Young and Well CRC) Abbotsford, VIC Australia

**Keywords:** MARS, mHealth, eHealth, app evaluation, end user, app trial, mhealth trial, user testing, mobile application, app rating, reliability, mobile health, well being, mental health, smartphone, cellphone, telemedicine, ehealth, emental health, e-therapy, Internet, online, cognitive behavioral therapy, anxiety, anxiety disorders, depression, depressive disorder, Australia, research translation, evidence-informed, mHealth implementation, mHealth evaluation, randomized controlled trial, RCT

## Abstract

**Background:**

The Mobile Application Rating Scale (MARS) provides a reliable method to assess the quality of mobile health (mHealth) apps. However, training and expertise in mHealth and the relevant health field is required to administer it.

**Objective:**

This study describes the development and reliability testing of an end-user version of the MARS (uMARS).

**Methods:**

The MARS was simplified and piloted with 13 young people to create the uMARS. The internal consistency and test-retest reliability of the uMARS was then examined in a second sample of 164 young people participating in a randomized controlled trial of a mHealth app. App ratings were collected using the uMARS at 1-, 3,- and 6-month follow up.

**Results:**

The uMARS had excellent internal consistency (alpha = .90), with high individual alphas for all subscales. The total score and subscales had good test-retest reliability over both 1-2 months and 3 months.

**Conclusions:**

The uMARS is a simple tool that can be reliably used by end-users to assess the quality of mHealth apps.

## Introduction

Mobile health (mHealth) apps have the potential to provide around-the-clock access to evidence-based health information, education, and treatment to end users on a global scale. There are currently more than 165,000 mHealth apps (free and paid) publicly available [1], yet the accuracy of the health information contained in these apps is not scrutinized by regulatory bodies [2], which could compromise user health and safety [3-5]. Concerns about the quality, efficacy, reliability, and security of mHealth apps are also often raised. While meta-analytic studies have demonstrated the efficacy of mHealth apps targeting physical activity and weight loss [6,7], the evidence base for other types of mHealth apps is poor at best [8-10].

In response to these issues, we developed the Mobile App Rating Scale (MARS) to provide researchers, professionals, and clinicians with a brief tool for classifying and assessing the quality of mHealth apps [11]. The 23-item MARS contains 4 objective quality subscales—engagement, functionality, aesthetics, and information quality—and a subjective quality rating. The MARS has demonstrated high levels of interrater reliability for evaluating the quality of mHealth apps on well-being [11] and mindfulness [9]. However, training and expertise in mHealth and the relevant health field is required to administer it. This paper describes the development and pilot testing of a simpler, *end user* version of the MARS (uMARS) and provides preliminary evidence for its internal consistency and test-retest reliability.

## Methods

### Study 1: Development and Pilot Testing of the uMARS

The original MARS was simplified through the following process. The professional version was first reviewed by 2 researchers to remove complex terminology from its items and response scales. Three items requiring professional expertise, pertaining to evidence base, app goals, and accuracy of app description, were removed. Readability of the MARS and the draft uMARS was then determined using the Flesch Reading Ease test [12,13], which has a score range of 0-100, with higher scores indicating easier readability. This measure also provides the estimated US school grade required for reading comprehension.

The draft uMARS was then pilot-tested with 13 young people, to ensure they understood the item content and response scales. The measure was embedded in prototype testing sessions of 2 mHealth apps: *Ray’s Night Out* [14] and *Music eScape* [15]. *Ray’s Night Out* uses a harm-minimization approach to increase young people’s alcohol knowledge and awareness of their drinking limits; *Music eScape* teaches young people how to identify and manage affect using music. Both are available on the iOS Apple app store.

Eligible participants were Australian residents aged 16 to 25 years, who had access to an iPhone 4 or later model. The *Ray’s Night Out* group comprised 1 male and 8 females with a mean age of 20.7 years (SD 1.6). The *Music eScape* group comprised 3 males and 1 female, with a mean age of 21.5 years (SD 1.9). After testing the apps and rating them with the uMARS scale, participants were asked the question “Do you have any comments or suggestions about the uMARS rating scale?” to identify any unclear or difficult items.

### Study 2: Testing the uMARS Internal Consistency and Test-Retest Reliability

The uMARS (Multimedia Appendix 1) provides a 20-item measure that includes 4 objective quality subscales—engagement, functionality, aesthetics, and information quality—and 1 subjective quality subscale.

The reliability of the uMARS was evaluated as part of a randomized controlled trial (RCT), testing the efficacy and quality of *Music eScape*. The RCT sample comprised 164 Australians aged 19.8 years on average (SD 2.51); 34 males. The highest level of education completed by 59.8% of the sample was secondary school, and 24.4% had completed a bachelor’s degree or higher. Most participants (57.9%) were students and 35.4% had full-time, part-time, or casual employment.

Participants were randomly allocated via a Web-based research management tool developed at the Queensland University of Technology to receive immediate or 1-month delayed access to the *Music eScape* app. Young people were asked to use the app as much as they liked over a month, using their own iPhones. The current iOS version at the time of the trial was iOS8. Participants received weekly text messages reminding them to do so. App ratings were collected using the uMARS at 1-, 3-, and 6-month follow-ups in the immediate access group. In the delayed access group, uMARS ratings were collected at 2-, 3-, and 6-month follow-ups (ie, after 1, 2, and 5 months of app access). At each assessment point, participants were asked if they had used the app since the last assessment, and only those who reported some use were included in analyses.

### Data Analysis

The internal consistencies of the uMARS subscales and total score were calculated using Cronbach's alpha. For the purpose of analysis, the “N/A” answer option for items 13-16 of the *information* subscale was recoded as “system missing,” as this option represents a qualitatively different response.

Test-retest reliabilities were calculated for the subscales and total scores of the uMARS after 1 month of app use and at 3 months post baseline (ie, a test-retest period of 1-2 months), and over 3 months (ie, between assessments at 3 and 6 months post baseline). Interclass correlation coefficients (ICCs) [16-18] were used, as they provide weighted values of rater agreement and assess proximity rather than equality of ratings. To calculate the ICCs, a random-effects average measures model with absolute agreement was utilized [16]. Data were analyzed with SPSS version 23 (SPSS Inc, Armonk, NY, USA).

## Results

### Study 1: Readability

Results of the Flesch-Kincaid readability tests are in Table 1. Scores indicated that the uMARS was written in plain English and that its required reading level was approximately grade 8.

### Pilot Participant Feedback

No suggestions for further scale improvement were made. Seven of the 13 participants who pilot-tested the scale left the comments or suggestions item blank, 4 wrote “no,” and 1 wrote “Well done. Good questions. Well explained.” Another wrote “I thought it was shorter/there is a brief or revised version of it?”

### Study 2: uMARS Internal Consistency

A total of 152 of the 164 (92%) participants completed the survey after 1 month of app use. Of these, 19 indicated they never used the app, and were excluded from analyses. For the remaining 133 participants, the total uMARS score had excellent internal consistency (Cronbach alpha = .90). Internal consistencies of its subscales were also very high (engagement alpha = .80; functionality alpha = .70; aesthetics alpha = .71; information alpha = .78; and satisfaction alpha = .78).

### uMARS Test-Retest Reliability

Test-retest reliabilities are presented in Table 2. A total of 113 participants completed the scale after 1 month of app use and at 3 months post baseline (ie, a test-retest period of 1-2 months), and 74 completed both the 3- and 6-month surveys (giving a 3-month test-retest period). All included participants had used the app at least once since the previous survey. The uMARS total score demonstrated *good* [16] levels of ICC of .66 and .70 over 1- to 2-month and 3-month periods, respectively. Levels for all subscales scores were similarly high.

**Table 1:** Readability ease and grade level scores of the original Mobile App Rating Scale and the simplified user version of the scale (uMARS).

MARS^a^ version	Reading ease	Readability level	Grade level	Reading age
Original	47.2	Difficult	9.5	15-16 years old
uMARS^a^	58.0	Plain English – fairly difficult	7.9	12-13 years old

^a^ MARS: Mobile App Rating Scale; uMARS: user version of the MARS.

**Table 2:** Test-retest reliability of the user version of the Mobile App Rating Scale (95% CI).

Subscale/item		1- to 2-month period (N=113)	3-month period (N=74)
**Engagement**		.71 (.66-.76)	.73 (.67-.78)
1	Entertainment	.60 (.41-.72)	.75 (.61-.85)
2	Interest	.69 (.55-.79)	.67 (.48-.79)
3	Customization	.61 (.44-.73)	.53 (.25-.70)
4	Interactivity	.55 (.35-.69)	.69 (.51-.81)
5	Target group	.72 (.59-.80)	.73 (.57-.83)
**Functionality**		.62 (.54-.68)	.69 (.61-.76)
6	Performance	.54 (.34-.69)	.71 (.53-.81)
7	Ease of use	.65 (.49-.76)	.72 (.55-.82)
8	Navigation	.62 (.45-.74)	.67 (.48-.79)
9	Gestural design	.61 (.44-.73)	.65 (.44-.78)
**Aesthetics**		.58 (.48-.66)	.68 (.59-.76)
10	Layout	.39 (.11-.58)	.48 (.18-.67)
11	Graphics	.70 (.56-.79)	.77 (.63-.85)
12	Visual appeal	.63 (.46-.75)	.80 (.68-.87)
**Information**		.48 (.38-.57)	.52 (.40-.62)
13	Quality of information	.48 (.24-.64)	.44 (.11-.65)
14	Quantity of information	.48 (.24-.64)	.32 (.08 to .57)
15	Visual information	.42 (.16-.60)	.75 (.61-.84)
16	Credibility of source	.51 (.29-.66)	.63 (.41-.77)
Total uMARS^a^		.66 (.63-.68)	.70 (.67-.78)
**Subjective items**		.70 (.64-.75)	.71 (.64-.77)
17	Would you recommend	.84 (.76-.89)	.75 (.60-.84)
18	How many times	.44 (.18-.61)	.48 (.17-.67)
19	Would you pay	.81 (.73-.87)	.82 (.71-.89)
20	Overall (star) rating	.71 (.59-.80)	.77 (.63-.85)

^a^ uMARS: user version of the Mobile App Rating Scale.

## Discussion

This study developed and tested an app user version of the original MARS to assist app developers and researchers with evaluating the quality of mHealth apps. The uMARS (Multimedia Appendix 1) provides a 20-item measure that includes 4 objective quality subscales—engagement, functionality, aesthetics, and information quality—and 1 subjective quality subscale. One further subscale, consisting of 6 items is added to measure users’ perceived impact of the evaluated app. The study demonstrated that the uMARS had excellent internal consistency for the full scale and good levels for all subscales. It is reassuring that even after a 3-month delay between ratings, test-retest reliability of the total score was good, and test-retest reliabilities of its subscales were *fair* to *good*, with the *engagement* and *subjective* subscales being particularly robust.

These results indicate that the uMARS provides a reliable measure of app quality in target users. Replication of the current results with multiple types of mHealth apps is required to provide additional confidence in its performance. Tests of its sensitivity to improvements in app quality and an examination of its ability to predict outcomes of mHealth apps are also needed. As the uMARS may potentially have applications beyond mHealth, tests of its performance in other domains are also indicated.

Current indications are that the uMARS will offer an unprecedented ability to readily obtain rich information from users about mobile apps. The scale can be used to obtain user feedback on the quality of mobile apps during the development and testing process, which may result in overall improvements in their quality.

## Appendix

Multimedia Appendix 1

## Abbreviations

ICC: interclass correlation coefficient
MARS: Mobile App Rating Scale
mHealth: mobile health
RCT: randomized controlled trial
