# Supplementary material for: Development and Validation of the User Version of the Mobile Application Rating Scale (uMARS)
Source: JMIR Mhealth Uhealth. 2016 Jun 10;4(2):e72. doi: 10.2196/mhealth.5849 (PMC4920963; doi:10.2196/mhealth.5849)
Supplement: Multimedia Appendix 1 [file mhealth_v4i2e72_app1.pdf]

Instructions for use:

Raters should:

1. Use the app and trial it thoroughly for at least 10 minutes;
2. Determine how easy it is to use, how well it functions and does it do what it purports to do;
3. Review app settings, developer information, external links, security features, etc.

Scoring

A: Engagement Mean Score = ________________

B: Functionality Mean Score = ________________

C: Aesthetics Mean Score = _________________

D: Information Mean Score* = ________________

* Exclude questions rated as "N/A" from the mean score calculation.

App quality mean score ___________________= A + B + C + D / 4

The *App subjective quality* scale can be reported as individual items or as a mean score, depending on the aims of the research.

The *Perceived impact* items can be adjusted and used to obtain information on the perceived impact of the app on the user's knowledge, attitudes and intentions related to the target health behaviour.

Mobile Application Rating Scale: user version (uMARS)

App Name: ____

Circle the number that most accurately represents the quality of the app you are rating. All items are rated on a 5-point scale from “1.Inadequate” to “5.Excellent”. Select N/A if the app component is irrelevant.

App Quality Ratings

SECTION A

Engagement – fun, interesting, customisable, interactive, has prompts (e.g. sends alerts, messages, reminders, feedback, enables sharing)

1. Entertainment: Is the app fun/entertaining to use? Does it have components that make it more fun than other similar apps?

1 Dull, not fun or entertaining at all
2 Mostly boring
3 OK, fun enough to entertain user for a brief time (< 5 minutes)
4 Moderately fun and entertaining, would entertain user for some time (5-10 minutes total)
5 Highly entertaining and fun, would stimulate repeat use

2. Interest: Is the app interesting to use? Does it present its information in an interesting way compared to other similar apps?

1 Not interesting at all
2 Mostly uninteresting
3 OK, neither interesting nor uninteresting; would engage user for a brief time (< 5 minutes)
4 Moderately interesting; would engage user for some time (5-10 minutes total)
5 Very interesting, would engage user in repeat use

3. Customisation: Does it allow you to customise the settings and preferences that you would like to (e.g. sound, content and notifications)?

1 Does not allow any customisation or requires setting to be input every time
2 Allows little customisation and that limits app’s functions
3 Basic customisation to function adequately
4 Allows numerous options for customisation
5 Allows complete tailoring the user’s characteristics/preferences, remembers all settings

4. Interactivity: Does it allow user input, provide feedback, contain prompts (reminders, sharing options, notifications, etc.)?

1 No interactive features and/or no response to user input
2 Some, but not enough interactive features which limits app’s functions
3 Basic interactive features to function adequately
4 Offers a variety of interactive features, feedback and user input options
5 Very high level of responsiveness through interactive features, feedback and user input options

5. **Target group: Is the app content (visuals, language, design) appropriate for the target audience?**

   1 Completely inappropriate, unclear or confusing
   2 Mostly inappropriate, unclear or confusing
   3 Acceptable but not specifically designed for the target audience. May be inappropriate/ unclear/confusing at times
   4 Designed for the target audience, with minor issues
   5 Designed specifically for the target audience, no issues found

SECTION B

Functionality – app functioning, easy to learn, navigation, flow logic, and gestural design of app

6. **Performance: How accurately/fast do the app features (functions) and components (buttons/menus) work?**

   1 App is broken; no/insufficient/inaccurate response (e.g. crashes/bugs/broken features, etc.)
   2 Some functions work, but lagging or contains major technical problems
   3 App works overall. Some technical problems need fixing, or is slow at times
   4 Mostly functional with minor/negligible problems
   5 Perfect/timely response; no technical bugs found, or contains a ‘loading time left’ indicator (if relevant)

7. **Ease of use: How easy is it to learn how to use the app; how clear are the menu labels, icons and instructions?**

   1 No/limited instructions; menu labels, icons are confusing; complicated
   2 Takes a lot of time or effort
   3 Takes some time or effort
   4 Easy to learn (or has clear instructions)
   5 Able to use app immediately; intuitive; simple (no instructions needed)

8. **Navigation: Does moving between screens make sense; Does app have all necessary links between screens?**

   1 No logical connection between screens at all /navigation is difficult
   2 Understandable after a lot of time/effort
   3 Understandable after some time/effort
   4 Easy to understand/navigate
   5 Perfectly logical, easy, clear and intuitive screen flow throughout, and/or has shortcuts

9. **Gestural design: Do taps/swipes/pinches/scrolls make sense? Are they consistent across all components/screens?**

   1 Completely inconsistent/confusing
   2 Often inconsistent/confusing
   3 OK with some inconsistencies/confusing elements
   4 Mostly consistent/intuitive with negligible problems
   5 Perfectly consistent and intuitive

SECTION C

Aesthetics – graphic design, overall visual appeal, colour scheme, and stylistic consistency

10. Layout: Is arrangement and size of buttons, icons, menus and content on the screen appropriate?

1 Very bad design, cluttered, some options impossible to select, locate, see or read
2 Bad design, random, unclear, some options difficult to select/locate/see/read
3 Satisfactory, few problems with selecting/locating/seeing/reading items
4 Mostly clear, able to select/locate/see/read items
5 Professional, simple, clear, orderly, logically organised

11. Graphics: How high is the quality/resolution of graphics used for buttons, icons, menus and content?

1 Graphics appear amateur, very poor visual design - disproportionate, stylistically inconsistent
2 Low quality/low resolution graphics; low quality visual design – disproportionate
3 Moderate quality graphics and visual design (generally consistent in style)
4 High quality/resolution graphics and visual design – mostly proportionate, consistent in style
5 Very high quality/resolution graphics and visual design - proportionate, consistent in style throughout

12. Visual appeal: How good does the app look?

1 Ugly, unpleasant to look at, poorly designed, clashing, mismatched colours
2 Bad – poorly designed, bad use of colour, visually boring
3 OK – average, neither pleasant, nor unpleasant
4 Pleasant – seamless graphics – consistent and professionally designed
5 Beautiful – very attractive, memorable, stands out; use of colour enhances app features/menus

SECTION D

Information – Contains high quality information (e.g. text, feedback, measures, references) from a credible source

13. Quality of information: Is app content correct, well written, and relevant to the goal/topic of the app?

N/A There is no information within the app
1 Irrelevant/inappropriate/incoherent/incorrect
2 Poor. Barely relevant/appropriate/coherent/may be incorrect
3 Moderately relevant/appropriate/coherent/and appears correct
4 Relevant/appropriate/coherent/correct
5 Highly relevant, appropriate, coherent, and correct

14. Quantity of information: Is the information within the app comprehensive but concise?

N/A There is no information within the app
1 Minimal or overwhelming
2 Insufficient or possibly overwhelming
3 OK but not comprehensive or concise
4 Offers a broad range of information, has some gaps or unnecessary detail; or has no links to more information and resources
5 Comprehensive and concise; contains links to more information and resources

15. **Visual information: Is visual explanation of concepts – through charts/graphs/images/videos, etc. – clear, logical, correct?**

N/A There is no visual information within the app (e.g. it only contains audio, or text)
1 Completely unclear/confusing/wrong or necessary but missing
2 Mostly unclear/confusing/wrong
3 OK but often unclear/confusing/wrong
4 Mostly clear/logical/correct with negligible issues
5 Perfectly clear/logical/correct

16. **Credibility of source: does the information within the app seem to come from a credible source?**

N/A There is no information within the app
1 Suspicious source
2 Lacks credibility
3 Not suspicious but legitimacy of source is unclear
4 Possibly comes from a legitimate source
5 Definitely comes from a legitimate/specialised source

App subjective quality

SECTION E

17. **Would you recommend this app to people who might benefit from it?**

1	Not at all	I would not recommend this app to anyone
2		There are very few people I would recommend this app to
3	Maybe	There are several people I would recommend this app to
4		There are many people I would recommend this app to
5	Definitely	I would recommend this app to everyone

18. **How many times do you think you would use this app in the next 12 months if it was relevant to you?**

1 None
2 1-2
3 3-10
4 10-50
5 >50

19. **Would you pay for this app?**

1 Definitely not
2
3
4
5 Definitely yes

20. **What is your overall (star) rating of the app?**

1	★	One of the worst apps I’ve used
2	★★	
3	★★★	Average
4	★★★★	
5	★★★★★	One of the best apps I've used

Perceived impact

SECTION F

1. **Awareness – This app has increased my awareness of the importance of addressing the health behaviour**

Strongly disagree				Strongly Agree
1	2	3	4	5

2. **Knowledge – This app has increased my knowledge/understanding of the health behaviour**

Strongly disagree				Strongly Agree
1	2	3	4	5

3. **Attitudes – The app has changed my attitudes toward improving this health behaviour**

Strongly disagree				Strongly Agree
1	2	3	4	5

4. **Intention to change – The app has increased my intentions/motivation to address this health behaviour**

Strongly disagree				Strongly Agree
1	2	3	4	5

5. **Help seeking – This app would encourage me to seek further help to address the health behaviour (if I needed it)**

Strongly disagree				Strongly Agree
1	2	3	4	5

6. **Behaviour change – Use of this app will increase/decrease the health behaviour**

Strongly disagree				Strongly Agree
1	2	3	4	5

Further comments about the app?

THANK YOU!
